# Communication

**Published:** 1901-05

**Authors:** Leonard Pearson

**Affiliations:** Philadelphia


### COMMUNICATION.

This letter is one of a series issued annually by the veterinarian of the Pennsylvania State Live-stock Sanitary Board, Dr. Leonard Pearson, and sent to the members of the profession in the State :

PHILADELPHIA, April 26, 1901.

DEAR DOCTOR : At the beginning of this letter I wish to state that it is sent to a large number of Pennsylvania veterinarians who are known to be interested in sanitary work and in the operations of the State Live-stock Sanitary Board. It is the third of a series. I mention this fact because a few of those who received the last circular letter regarded it as an individual communication, and thought that the statements in respect to the fraudulent inspections of Inter-State cattle were intended as personal reflections upon them.

Please do not fail to note Section 10, to which a reply is desired.

1. *Another Inspector Dropped.* Since the preparation of the last circular letter yet another inspector has been dropped from the list of approved veterinarians, and no more reports from him can be accepted on account of the fraudulent character of his work.

2. *The special course for veterinarians* that was held at the veterinary department of the University of Pennsylvania during the week in which the meeting of the State Veterinary Society was held attracted a large attendance. Unfortunately, it became necessary during the course to make a pilgrimage to Harrisburg to appear before the Committee on Public Health and Sanitation in opposition to the Stiles' bill. After this hearing, on Thursday evening, a large number of the members of the special course did not return to Philadelphia but continued on their way home. This considerably reduced the attendance during the last days of the course, but still the work was entirely satisfactory to those who remained and to the instructors..

The meeting of the State Veterinary Society was one of the largest and best in the history of the organization. It appears, however, that it is not desirable to have the special course and the meeting of the State Society during the same week. Next year the original plan will be returned to, where under the special course will precede or follow the meeting of the State Society. The papers read at the recent State meeting, together with the discussions and resolutions, will be printed in the JOURNAL OF COM-

PARATIVE MEDICINE AND VETERINARY ARCHIVES. The banquet given by Dr. Hoskins was a most enjoyable feature of the week. This was also the occasion of the presentation of a silver service to Dr. Hoskins by the veterinarians of the State in token of their appreciation of his services to the profession.

3. *The Proposition to Open the Veterinary Registration Law Has Been Defeated.* This is a direct result of the influence of the veterinary profession of the State brought to bear upon the Legislature. It has been most gratifying and encouraging to note the vast change that has occurred during the past few years in the attitude of the Legislature (and, indeed, the whole public) toward the veterinary profession. This is due to improvements in the general personnel of the profession and also to better organization, rendering it possible for the individuals to work together and exert all of their power in a well-ordered and well-planned way. The Legislative Committee of the State Veterinary Society is to be congratulated on their success in this matter.

I believe that no harm would have come, and, on the whole, it would have been well to have so amended the law that men who could show to the State Examining Board that they were eligible to register in 1899 should now be permitted to register. This is the view taken by the State Examining Board, and if adopted would perhaps have had a tendency to stop attempts to amend the law in the future, but when the members of the Legislature perceived so many urgent requests to defeat the law they did so on the first opportunity.

4. *Inspection of Tuberculous Herds.* The applications for herd inspections have been more numerous of late than ever before. This is due no doubt to a fuller appreciation of the advantages of such inspections by herd owners. The applications have become so numerous that it is quite impossible for the Board to comply with all of them, and much disappointment results. It has not heretofore been possible to obtain more than $40,000 a year for the general work of the Live-stock Sanitary Board, and this sum is not now sufficient to do the work that the Board is asked to do. One of two things must be done: more money must be obtained for this work or the plan of operation must be recast in such a way as to continue to encourage herd owners to suppress tuberculosis among their cattle, but to bear themselves a greater proportion of the expense and loss. The former plan does not appear to be feasible, and, indeed, $40,000 a year ought to be as much of the loss from tuberculosis and other diseases as the State should be required to bear directly from its treasury. The State cannot be expected to conduct a general insurance operation and compensate the owners of sick or dead cattle indiscriminately for their losses. This is not a proper function of State Government.

It appears, however, to be the duty of the State to protect herd owners from dangers that they cannot protect themselves against, and also to pay for private property when it is necessary to condemn such property for the protection of the public. Now that so much work on tuberculosis has been done and the subject has been discussed so fully, should the State continue to compensate herd owners for the cattle that they deliberately allow to become infected by disregarding precautions widely known and thoroughly tested? I believe that payments for tubercular cattle should continue for a number of years, but that inspections at public expense should henceforth be surrounded by greater restrictions in order to more effectually secure the permanency of the work that is done. A step in this direction would consist in requiring that all owners of herds tested henceforth by the State shall add to such herds no cattle that are not shown by tuberculin test to be free from disease. It might also be required that the herd owner shall, at his own expense, have a retest made within a certain time after the original inspection.

I should like to have a brief statement of your views in regard to this whole subject.

5. *Farmers' Institutes.* The veterinarians can do a great deal for the farmers of Pennsylvania by joining and attending the meetings of farmers' clubs and other organizations and institutes. The veterinarians should be the natural and leading experts in their communities on the more important questions pertaining to animal husbandry. Veterinarians are in a position to encourage the keepers of good stock, and they can do much to favor a grading up of all farm animals. It is a notorious fact that the average farm animal does not yield in product or work or selling value more than one-half as much as could be produced if the animal were bred better and fed and cared for in a better way. Veterinarians can do a great deal of good by pointing out the profitable types of animals to breed, and by furnishing instruction in regard to hygienic care, feeding, etc. This will do something to lessen the general prevalence of disease, but it will increase the value of the individual animal and will increase the owner's attention to it so that the practice of the veterinarian will gain instead of suffer.

Veterinarians who propose to engage in this work should remember that a great many able men are now engaged in the Farmers' Institute work of the various States, and farmers are accustomed to hearing and to discussing thorough, accurate, substantial addresses. I have known veterinarians who have failed in Farmers' Institute work because they assumed in the discussion of certain questions an attitude of superiority that they did not have the actual knowledge to support.

The speaker should always have reliable data and his facts well in hand.

Another factor that detracts from the influence of a veterinarian's talk before a body of farmers is the tendency to take an extreme position and to recommend things which are not regarded as feasible. Such a speaker is set down as a theoretical vaporizer, and his words go for naught.

6. *Rabies.* There has been a good deal of rabies in different parts of the State during the present spring. A number of cattle bitten by rabid dogs have developed rabies. On one farm in Chester County during the past winter twelve cows died of this disease. In this case the diagnosis was amply proven by inoculation.

The new method of diagnosis by microscopical examination of the cervical ganglia that has been developed in the United States by Dr. M. P. Ravenel is proving to be very satisfactory. This rapid method of diagnosis makes it possible to arrive at a definite conclusion in regard to the presence or absence of rabies of an animal under post-mortem examination within a short time, instead of having to wait several weeks as when resort is made to inoculation.

7. *Anthrax.* The hot weather that is conducive to the propagation of anthrax will soon be upon us.

I wish to again draw attention to the importance of not making post-mortem examinations on animals that there is reason to believe have died of this disease. By opening an anthrax carcass the operator is seriously exposed, oxygen is admitted to contact with the blood, the bacilli develop resisting spores, and the soil in the vicinity becomes permanently infected. It is much better to burn the carcass and have done with it, reserving only an ear cut off close to the head for shipment to the laboratory for bacteriological examination. The shipment should only be made in accordance with the directions heretofore given.

8. *Inter-State Cattle Inspection.* In accordance with the recommendation of the veterinarians in attendance upon the final session of the special course, the State Live-stock Sanitary Board has resolved to require affidavit to be made to reports on the inspection of Inter-State cattle. This is done for the purpose of protecting honest veterinarians against men who might do fraudulent work. If a man renders a false report he can be dropped from the list of approved inspectors; if a man swears to a false report he can be prosecuted and subjected to a penalty for perjury. It is to be hoped that wherever there is any reason to doubt the honesty of an Inter-State cattle inspector report of the fact will at once be made to me, so that the matter can be investigated and appropriate action taken. Irregular and false inspections will not be tolerated. If the veterinary profession of this State cannot be relied upon to properly inspect and test Inter-State cattle, the law had best be repealed.

Although a good deal has been said about fraudulent work in this connection and many reports of alleged frauds have been investigated, I am firmly of the opinion that on the whole the work has been done honestly and well. But the exceptional cases in which the work is not done properly are the ones we must trace to their origin and expose. I believe that the veterinarians of the State will not countenance inspections of a character that throw a cloud upon the entire profession.

9. *The bulletin of tuberculosis* that has been in preparation for some time is about to issue from the press and a copy will be mailed you.

10. *The laboratory work of the State Live-stock Sanitary Board* is the work upon which advancement chiefly depends. It is here that new methods are tried and old methods are tested and improved, difficult diagnosis made, new diseases studied, and biological products prepared for veterinarians of the State. This work is supported by a special appropriation, and it is very important to the live-stock sanitary interests of the State that this appropriation shall be continued, and, if possible, increased over that of last year, when it was cut down to $8000 ($4000 a year for two years). You will be interested to know that but one other laboratory in the United States has done as much original work on the diseases of animals as this one, and that laboratory costs six times as much to maintain. As I know that you appreciate the importance of this work, may I ask you to write to your Representative and to your Senator at Harrisburg requesting them to favor the proposed appropriation of $12,000 ($6000 a year for two years) for investigations concerning the diseases of domestic animals?

Will you kindly inform me what you have done in this matter—that is, to whom you have written—and also give me your views in regard to Section 14, and a report in reference to rabies in your vicinity.

Very truly yours,

LEONARD PEARSON.

